# Characteristics of a Spray-Dried Porcine Blood Meal for *Aedes aegypti* Mosquitoes

**DOI:** 10.3390/insects15090716

**Published:** 2024-09-19

**Authors:** Alexander R. Weaver, Nagarajan R. Rajagopal, Roberto M. Pereira, Philip G. Koehler, Andrew J. MacIntosh, Rebecca W. Baldwin, Christopher D. Batich

**Affiliations:** 1 Department of Chemical Engineering, University of Florida, 1030 Center Dr., Gainesville, FL 32611, USA; alrweaver02@gmail.com; 2 Department of Materials Science and Engineering, University of Florida, 549 Gale Lemerand Drive, P.O. Box 11400, Gainesville, FL 32611, USA; nagarajanrrajago@ufl.edu; 3 Entomology & Nematology Department, University of Florida, 1881 Natural Area Dr., Gainesville, FL 32611, USA; rpereira@ufl.edu (R.M.P.); pgk@ufl.edu (P.G.K.); baldwinr@ufl.edu (R.W.B.); 4 Department of Food Science and Human Nutrition, University of Florida, 572 Newell Dr., Gainesville, FL 32611, USA; andrewmacintosh@ufl.edu

**Keywords:** artificial meal, alternative meal, blood feeding, phagostimulant, *Aedes aegypti*, rearing, hemoglobin, spray drying

## Abstract

**Simple Summary:**

Mosquito vector research and population control strategies are supported by the rearing of species like *Aedes aegypti*, commonly known for propagating yellow fever. Colonies of *Ae. aegypti* are typically reared on fresh animal bloods to supply nutrients including iron, sodium chloride, and albumin necessary for reproduction. However, fresh animal blood is difficult to handle, store, and transport, and its use in rearing is limited to two weeks post-collection. The present study reports on the identification of an inexpensive, shelf-stable spray-dried porcine blood (SDPB) product containing accessible hemoglobin that demonstrated usefulness in a simple mosquito alternative meal (AM) formulation. Quantitative evidence that the phagostimulant in SDPB is hemoglobin was obtained through chromatography assays and the elimination of other feeding stimulants as potential candidates. *Ae. aegypti* observably engorged upon and produced eggs when fed the AM, which was also shown to sustain multiple colony generations with offspring viable through the larval and pupal stages. SDPB-based AMs may develop into platforms for studying the rearing of hematophagous insects with the potential to expand the capabilities of resource-intensive population control strategies.

**Abstract:**

Research into mosquito-borne illnesses faces hurdles because feeding fresh animal blood to rear female mosquitoes presents logistical, economic, and safety challenges. In this study, a shelf-stable additive (spray-dried porcine blood; SDPB) hypothesized to supply accessible hemoglobin was evaluated within an alternative meal (AM) containing whey powder and PBS for rearing the yellow fever mosquito *Aedes aegypti*. LC–MS/MS proteomics, microbial assays, and particle reduction techniques confirmed and characterized the functionality of hemoglobin in SDPB, while engorgement, fecundity, egg viability, and meal stability bioassays assessed AM performance. Chemical assays supported hemoglobin as the phagostimulant in SDPB with aggregates partially solubilized in the AM that can be more accessible via particle reduction. Unpaired two-tailed *t*-tests indicate that the AM stimulates oogenesis (t_11_ = 13.6, *p* = 0.003) and is stable under ambient (1+ y; t_12_ = 0.576, *p* = 0.575) and aqueous (14 d; t_12_ = 0.515, *p* = 0.639) conditions without decreasing fecundity. Egg hatch rates for the ninth generation of AM-reared *Ae. aegypti* were 50–70+%. With further development, this meal may serve as a platform for mass rearing or studying effects of nutritional additives on mosquito fitness due to its low cost and stability. Future work may examine tuning spray drying parameters and resulting impacts on hemoglobin agglomeration and feeding.

## 1. Introduction

The mosquito vector *Aedes aegypti* (L.) (Diptera: Culicidae) is known to transmit the infectious agents responsible for dengue, yellow fever, chikungunya, and Zika fever. The World Health Organization estimated in 2020 that vector-borne illnesses account for more than 500 million cases and 700,000+ deaths globally per year [1]. Colonies of vectors such as *Ae. aegypti* are both mass-reared for population control schemes and on a smaller scale in research laboratories for this reason. Under all circumstances, female mosquitoes require a meal, typically vertebrate blood, to reproduce. It is traditionally presented in one of two ways: either by using a live host [2,3] (i.e., via the skin of a live animal) or through an artificial or biologically-derived membrane [4,5,6] (i.e., stored defibrinated blood fed through PFTE or Parafilm^®^). Feeding fresh blood remains the standard as defibrinated bovine, monkey, and rabbit blood [7], among other types, are accessible for purchase in certain parts of the world.

There are several challenges associated with feeding fresh defibrinated blood to mosquitoes. The use of defibrinated blood poses issues of availability, transportation, variability, presence of infectious agents, regulation, and storage [8,9,10]. It also possesses a refrigerated shelf life of only two weeks before spoiling and negatively affecting feeding [7,8], requiring those who continuously rear mosquitoes to purchase fresh blood biweekly. Frozen, citrated blood has been effectively used in mosquito rearing but does not circumvent biological risks and cold storage requirements [11].

An innovative solution to overcome current problems consists of developing a feeding substitute (alternative meal; AM) with the nutritional qualities of fresh blood at a comparable or lower cost. Gonzalez et al. have developed SkitoSnack, an alternative meal consisting primarily of bovine serum albumin, bovine hemoglobin, chicken egg yolk, adenosine triphosphate (ATP), and glucose in a salt buffer solution [8,9,12]. Hemoglobin of bovine origin and ATP are the main constituents of such AMs responsible for high cost and cold storage needs. Heme iron, contributed by its carrier protein hemoglobin, is the key source of the essential element for *Ae. aegypti* (98%) and the development of their eggs (97%) relative to ferric iron [12,13], while ATP acts as a phagostimulant to divert ingested meals to the midgut [8,14]. The costs of biochemical isolates such as hemoglobin, ATP, and albumin are driven by expensive purification steps, causing such alternative meals’ costs to approach or exceed that of fresh blood [9]. The key advantages of an alternative meal platform, especially for commercial applications, should be low cost and shelf stability.

Spray drying, an evaporative process driven by exposing small droplets of a solution to a hot gas [15,16], demonstrates utility as a preservation method for blood nutrients useful in an alternative meal [17,18] due to the short times in which they are exposed to heat and subject to degradation. To understand this phenomenon, the maximum wet-bulb temperature (T_wb_) of drying can be related to the inlet gas temperature (T_in_) and the boiling point of the solution (T_b_) (K):

T_wb_ = (137 × (T_b_/373.15)^0.68^ × log(T_in_)) − 45 (1)

Equation (1) empirically describes the evaporation of water from an aqueous droplet of particulate for a perfectly-tuned spray drying process [19]. In such a process, the dried product exits the drying chamber just as all water evaporates. It is known that human methemoglobin and oxyhemoglobin denature at 67 °C and 71 °C [20], respectively, so to achieve either denaturation temperature while aqueous, T_in_ must be 374 and 419 °C. These values of T_in_ are outside the bounds of blood spray drying applications cited in the literature (125 °C to 225 °C) [15,18,21,22,23]. Because the product’s exposure to heat can practically be brief, it is then possible that some functional hemoglobin is preserved through spray drying when drying gases are heated to less than 225 °C. Such protein preservation has been observed in animal bloods [17,21], spray-dried pharmaceuticals [19,24], and other protein solutions [18,23,25,26,27]. As an alternative to mixing individual components within a formulated meal, reconstituted spray-dried whole blood deserves examination as a cost-effective, shelf-stable variant of its fresh analogue for its ability to provide hemoglobin without the use of separations.

Because of the challenges imposed by using fresh bovine blood in *Ae. aegypti* rearing, the present study investigates the functional and material properties of spray-dried porcine blood and its use in a simple alternative meal. The dried blood product was principally evaluated on the hypothesis that hemoglobin is bioavailable to mosquitoes when SPDB is reconstituted. Second, a meal comprising the dried blood product and whey protein powder (primarily lactalbumin) in saline was used to measure the feeding impacts of materially altering SPDB. This formulation was lastly assessed for its effects on mosquito reproduction and egg viability, as well as its cost and storage requirements, from the critical perspective of its usefulness as a rearing platform.

## 2. Materials and Methods

Orlando-strain *Ae. aegypti* mosquitoes were used in all bioassays and came from two sources: (1) a defibrinated bovine blood-fed line maintained in the University of Florida’s Urban Entomology laboratory, and (2) pupae sourced from the CMAVE—USDA (Center for Medical, Agricultural, and Veterinary Entomology at the U.S. Department of Agriculture) in Gainesville, FL, USA, also used by Tyler-Julian et al. [11]. Mosquitoes were collected and reared weekly. For the former source, using a vacuum apparatus, *Ae. aegypti* eggs were stimulated to hatch in a deli cup containing well water for 30 min and subsequently transferred to a larger rearing container at 28 °C and 70% relative humidity. Larvae were fed pulverized goldfish food (TetraMin Plus Tropical Flakes, Tetra GMBH, Melle, Germany) for one week [2,28]. Pupae were moved in a plastic cup to a BugDorm-1© (MegaView Science Co., Ltd., Taiwan, Republic of China) mosquito-rearing cage (0.027 m^3^) with three screened sides and three clear plastic sides. BugDorm-1© cages contained a cup with 10% sucrose (*w*/*v*) in water [28] and a lid with a cotton wick so that emerging adults had access to the sugar solution. Mosquito cages were stored in a 28 °C rearing room at 70% RH [2] on a 12-h photoperiod. Adult *Ae. aegypti* aged 6–15 d post-emergence were used in all experiments. The pesticide-susceptible Orlando strain used in the experiments was maintained with fresh (less than two weeks old) defibrinated bovine blood sourced from the CMAVE—USDA. Eggs laid on moistened, then desiccated, germination papers were used to hatch subsequent generations of larvae [28].

### 2.1. AM Formulation and Preparation

All alternative meals were prepared in a 1× buffer solution of Thermo Scientific 10X PBS (pH 7.4, catalog no. J62036.K3; Waltham, MA, USA) and deionized water, stirred magnetically at 250 rpm. Phosphate buffers mimic physiological conditions and are known to stabilize albumin; they were diluted to avoid destabilizing hemoglobin [29]. Preparation of the simple AM is described in Table 1. Unless otherwise noted, AMs used in experimentation were prepared as described below (“simple” formulation).

Spray-dried porcine blood (SDPB, sourced from Earthworks Health LLC, Norfolk, NE, USA and Tyson Ingredient Solutions, Springdale, AR, USA) was reconstituted at a concentration of 20% (*w*/*v*) in 1× PBS to mimic the 17–18% concentration of animal proteins in blood [30]. Then, 10% whey protein powder was added as an albumin source (*w*/*v*; lot # UH091 and 67963G22; Jarrow Formulas, Sherman Oaks, CA, USA). The undiluted product was then used in feeding. When allowed to settle, SDPB naturally segregates into three distinct layers, so the powder was ground using a handheld coffee grinder. A wide-tip pipette was used to transfer 2.5 mL of agitated AM or fresh bovine blood.

**Figure 1:**
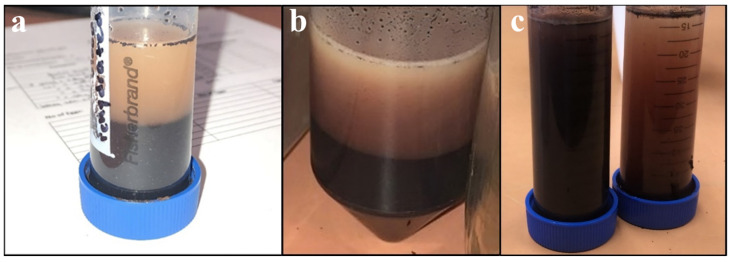
Aqueous preparations of (**a**) the simple AM with unground SDPB after complete segregation, (**b**) the simple AM after complete segregation, and (**c**) espresso-ground SDPB (**left**) and unground SDPB (**right**) upon sitting for several minutes.

Due to the relatively large distribution of particle sizes present in unground SDPB, the powder was ground using one of three methods pertaining to experimental objectives: coffee-grinding (using a motor-driven handheld unit), espresso-grinding (using a finer mechanical unit), or ball milling. Ball milling was conducted with 5 mm yttria-stabilized zirconia (YSZ) media at 300 rpm for varying amounts of time. Figure 1 shows segregation of (a) unground, (b) coffee-ground, and (c) espresso-ground aqueous preparations including SDPB.

Unless otherwise specified, all AMs contained SDPB ground with a handheld coffee grinder.

### 2.2. General Feeding Bioassay

To obtain fecundity data, adult *Ae. aegypti* were transferred from BugDorm-1© cages to feeding cups (plastic 7 × 10 cm containers containing a 3 × 5 cm souffle cup with a germination paper strip and a 3 × 3.5 cm deli cup with a cotton ball for sucrose feeding) using a mechanical aspirator. Feeding cups were covered with plastic mesh. Ten to twenty female mosquitoes were placed in each cup and counted.

The meal feeding apparatus for all controls and experimental AMs consisted of Sigma Scientific (12109 US-441, Micanopy, FL, USA) 3.4 cm bottom diameter glass thermal jackets with Parafilm^®^M, which is commonly used as an artificial feeding membrane for insects [5], thinly stretched over their exposed ends using ungloved hands similar to the heated plate technique used by Tyler-Julian et al. [11]. The glass feeders were connected by plastic tubing to an isothermal bath maintaining 36–38 °C water [4,7] and a peristaltic pump feeding the jackets at 300 mL/min. The use of thermal jackets as compared to the heated surfaces used by Tyler-Julian et al. allowed for feeding of multiple enclosures within a controlled temperature range. Minor temperature differences across the feeding chain were minimized to ≤1 °C by distributing the feeders along two parallel chains. Environmental conditions were set to 23 ± 2 °C and 55 ± 5% RH maintained by a building HVAC system. Feeding cups (120 mL plastic cups) with 10–20 mosquitoes (6–15 d old) were placed under Parafilm-covered thermal jackets similar to the devices constructed by Luo and Pothikasikorn et al. [4,7] for 2 h. The low density of mosquitoes in feeding enclosures allowed for easy observation and accurate counting. The cups were then removed from the feeding apparatus and mosquitoes were assessed for engorgement.

Feeding cups were provided with 10% sucrose solution in tap water via a soaked cotton ball to support oogenesis [2]. Then, 3.8 × 3.8 cm germination papers soaked in 25 mL of well water served as egg-laying surfaces similar to the method used by Imam et al. [2]. All cups were placed in a mosquito-rearing room for an additional 7 d before eggs were counted.

To count eggs, feeding enclosures were placed in a freezer for 15+ min to euthanize mosquitoes. Enclosures were then disassembled and cleaned, and their germination papers were removed. Germination papers were left to dry for 7 d at room temperature, and viable eggs (unfragmented and of the proper ovular shape) were counted under a dissecting microscope.

### 2.3. Experimental Design

A negative control (i.e., an AM lacking SDPB or whey) and/or a positive fresh bovine blood control were included in all feeding trials to provide comparisons with AMs. Unless otherwise specified, fresh defibrinated bovine blood was sourced from the CMAVE—USDA. Parallel trials were conducted at the Entomology & Nematology Department, University of Florida (1881 Natural Area Dr., Gainesville, FL, USA), with active female mosquitoes (demonstrating the ability to probe at a heat source) of age 5–15 d meeting eligibility criteria. Feeding bioassays were arranged such that individual cages alternated between experimental meals along a feeding chain, and the linear arrangement of cages was randomized by female mosquito count before assignment to feeders to ensure that conditions received approximately the same total number of mosquitoes. The number of replicates used reflected the maximum number of feeders available on a given day and the ability of the water bath to maintain the specified temperature of 36–38 °C across a chain.

All mosquitoes used in fecundity assays were starved of sucrose solution for 2 h prior to feeding. Feeding was performed for 2 h in all experiments. Fewer replicates were used for experiments not measuring fecundity than those measuring fecundity due to the lower variability in engorgement on the AM between individual mosquitoes.

Obtaining repeatable results for mosquito feeding is challenging, so the use of multiple replicate enclosures in feeding assays ensured that data were sufficiently representative. For example, in an experimental condition with three replicate enclosures, a minimum of 45 mosquitoes were present; with eight replicate enclosures, a minimum of 120 mosquitoes were present. Average fecundity was the outcome metric of most bioassays and was calculated by dividing the total number of viable eggs laid by the number of female mosquitoes counted within an enclosure.

### 2.4. Phagostimulant Identification

#### 2.4.1. SEM/Light Microscopy and Laser Diffraction

The ability of mosquitoes to ingest nutritive molecules is primarily dictated by the individual diameters of nutrient particles, so microscale visual inspection of SDPB particles using scanning electron microscopy (SEM) was performed to estimate the particle diameter distribution of unground and ground SDPB powders. Imaging was performed with a Thermo Scientific Phenom™ XL G2 (Thermo Fischer Scientific, Waltham, MA, USA) desktop unit. SDPB was scattered across carbon tape, and pressurized air was used to remove particles over 1 mm in diameter to avoid damaging the scanning microscope. After coating with Au–Pd, particles were scanned at 5 kV. Particle diameter processing was performed digitally within the Thermo Scientific Phenom™ ProSuite application.

Three SDPB preparations were prepared and imaged: unground, espresso-ground, and espresso-ground sieved through a 63 μm mesh. Sieving was performed by shaking espresso-ground SDPB over the 63 μm mesh for 10 min. Twenty particles in images of espresso-ground and sieved SDPB were randomly selected, and their diameters were digitally computed and counted.

Light microscopy of SDPB particulate was performed to gain insight into particle morphology and color detail omitted in SEM. A Nikon Eclipse Ci-L Plus LED-illuminated unit (Nikon Instruments Inc., Melville, NY, USA) was used to image particulate suspended in Type A immersion oil (Cargille Laboratories, Cedar Grove, NJ, USA) under 10× and 40× magnification. Particulate suspensions were created on microscope slides etched with Neubauer hemocytometer squares of side lengths 0.1 mm and 0.05 mm.

Solutions of 20% unground SDPB, 20% ground SDPB, and 10% whey protein powder (all *w*/*v*) were individually prepared using deionized water and counted using a Beckman Coulter LS 13 320 laser diffraction particle counter. Particle analysis was performed at the University of Florida Research Service Center (Gainesville, FL, USA). Additional measurements were taken for dry, unground, and unhomogenized SDPB. Error bars on graphed data represent standard deviation.

#### 2.4.2. LC–MS/MS Proteomics

To assemble a list of phagostimulant candidates from identifiable proteins in SDPB, LC–MS/MS proteomics were performed at the University of Florida Department of Chemistry Mass Spectrometry Research and Education Center (MSREC; Gainesville, FL, USA). Unground SDPB, coffee-ground SDPB, ball-milled SDPB (milled for 72 h), and whey protein powder were analyzed. Proteins were extracted and digested using the EasyPep™ MS Sample Prep Kit (Thermo Fisher Scientific, Waltham, MA, USA). Total protein was determined on a Qubit and the appropriate volume of each sample was taken to equal 20 µg total protein for digestion. The samples were digested with a sequencing grade trypsin/lys C rapid digestion kit from Promega (Madison, WI, USA) using manufacturer-recommended protocols. Three times the sample volume of rapid digestion buffer (provided with the kit) was added to the samples. The sample was incubated at 56 °C with 1 µL of dithiothreitol (DTT) solution (0.1 M in 100 mM ammonium bicarbonate) for 30 min prior to the addition of 0.54 µL of 55 mM iodoacetamide in 100 mM ammonium bicarbonate. Iodoacetamide was incubated at room temperature in darkness for 30 min. Trypsin/lys C was prepared fresh as 1 µg/µL in the rapid digestion buffer. Then, 1 µL of enzyme was added and the samples were incubated at 70 °C for 1 h. The digestion was stopped with the addition of 0.5% TFA. MS analysis was immediately performed to ensure high quality tryptic peptides with minimal nonspecific cleavage.

Nano-liquid chromatography tandem mass spectrometry (Nano-LC/MS/MS) was performed on a Thermo Scientific Q Exactive HF Orbitrap mass spectrometer equipped with an EASY Spray nanospray source (Thermo Scientific) operated in positive ion mode. The LC system was an UltiMate™ 3000 RSLCnano system from Thermo Scientific. The mobile phase A was water containing 0.1% formic acid, and the mobile phase B was acetonitrile with 0.1% formic acid. The mobile phase A for the loading pump was water containing 0.1% trifluoracetic acid. Then, 5 µL of sample was injected on to a PharmaFluidics µPAC C18 trapping column (5 mm pillar diameter, 10 mm length, 2.5 µm interpillar distance) at 10 µL/mL. This was held for 3 min and washed with 1% B to desalt and concentrate the peptides. The injector port was switched to injection mode, and the peptides were eluted from the trap and onto the column. PharmaFluidics 50 cm µPAC (Thermo Fisher Scientific B.V.B.A., Zaventem, Belgium) was used for chromatographic separations (C18, 5 µm pillar diameter, 50 cm length, 2.5 µm interpillar distance). The column temperature was maintained at 40 °C. A flowrate of 750 nL/min was used for the first 15 min, and then the flow was reduced to 300 nL/min. Peptides were eluted directly off the column into the Q Exactive system using a gradient of 1% B to 20% B over 100 min and then to 45% B in 20 min for a total run time of 150 min. Elution media concentrations and flow rates with respect to time are available in Supplementary Table S1.

The MS/MS was acquired according to standard conditions established at the University of Florida MSREC. The EASY Spray source was operated with a spray voltage of 1.5 KV and a capillary temperature of 200 °C. The scan sequence of the mass spectrometer was based on the original TopTen™ method; the analysis was programmed for a full scan recorded between 375 and 1575 Da at 60,000 resolution, and an MS/MS scan at resolution 15,000 to generate product ion spectra to determine amino acid sequence in consecutive instrument scans of the fifteen most abundant peaks in the spectrum. The AGC Target ion number was set at 3 × 10^6^ ions for full scan and 2 × 10^5^ ions for MS^2^ mode. Maximum ion injection time was set at 50 ms for full scan and 55 ms for MS^2^ mode. Microscan number was set at 1 for both full scan and MS^2^ scan. The HCD fragmentation energy (N)CE/stepped NCE was set to 28 with an isolation window of 4 m/z. Singly-charged ions were excluded from MS^2^. Dynamic exclusion was enabled with a repeat count of 1 within 15 s and to exclude isotopes. A siloxane background peak at 445.12003 was used as the internal lock mass.

The HeLa protein digest standard was used to evaluate the integrity and the performance of the columns and mass spectrometer. If the number of protein IDs from the HeLa standard fell below 3200, the instrument was cleaned, and new columns were installed.

All MS/MS spectra were analyzed using Sequest (Thermo Fisher Scientific, San Jose, CA, USA; version IseNode in Proteome Discoverer 3.0.1.27). Sequest was set up to search *Sus scrofa domesticus* (NcbiAV TaxID = 9825) assuming the digestion enzyme trypsin. Sequest was searched with a fragment ion mass tolerance of 0.020 Da and a parent ion tolerance of 10.0 ppm. Carbamidomethyl of cysteine was specified in Sequest as a fixed modification. Met-loss of methionine, met-loss + acetyl of methionine, and oxidation of methionine and acetyl of the n-terminus were specified in Sequest as variable modifications.

#### 2.4.3. ATP Assays and Microbial Contamination

The presence of ATP in an aqueous preparation can be detected in minute concentrations and/or indicate microbial contamination. For this reason, ATP hydrolysis via luciferin/luciferinase in various solutions was quantitatively measured via Hygenia^®^ Ensure^®^ luminescence measurements (Hygenia LLC, Camarillo, CA, USA) using Hygenia^®^ AquaSnap Total ATP^®^ swabs with a minimum of triplicate measurements taken per Hygenia’s testing procedure [31,32]. Unpaired two-tailed *t*-tests were used to compare all conditions subjected to AquaSnap Total ATP^®^ tests. Error bars on graphed data represent standard deviation.

To assess whether microbial contamination could be stimulating the engorgement of SDPB by contributing ingestible ATP to AMs, unground SDPB was plated on tryptic agar and incubated to indicate the presence of microbes. Then, 40 g of BD™ Bacto™ tryptic soy broth (soybean–casein digest medium, USP, ref. # 211825, lot # 2030828; Becton, Dickinson, & Co., Franklin Lakes, NJ, USA) was added to 1 L of deionized water, boiled, and cooled briefly before plating. Boiled and deionized (BDI) water, unground SDPB autoclaved at 121 °C for 60 min in BDI water, SDPB from an unopened package in BDI water, and EC-1118 *Saccharomyces cerevisiae* (Lalvin; Edwardstown, SA, Canada) in BDI water were individually plated with five replicates of each condition in a positive-pressure aseptic filling hood. ATP assays were conducted for each aqueous preparation after filling with a minimum of triplicate measurements taken. Incubation of cultures was performed at 28 °C for 18 h.

#### 2.4.4. Heat Sterilization

To understand whether a contaminant metabolite (i.e., ATP) or a native SDPB component was the primary driver of AM engorgement, mosquitoes were fed the simple AM containing either unground SDPB from an unopened package (in theory, uncontaminated) or unground SDPB autoclaved at 121 °C for 60 min. All preparatory materials used to handle the AM were aseptically cleaned with 95% EtOH before use. ATP assays were performed immediately after preparation (0 min) and after feeding (2 h) to validate the low microbial load of autoclaved SDPB and track microbial growth over the course of a feeding period. Feeding was performed with four replicates of each condition and according to the provided general feeding experimental design. An unpaired two-tailed *t*-test was used to compare the two conditions. Error bars on graphed data represent standard error.

#### 2.4.5. Ball Milling

It was hypothesized that aggregates of hemoglobin could be made more accessible via particle size reduction to improve feeding, so SDPB was ground for 72 and 96 h via ball milling and were each fed within the simple AM. Separate controls were required as 72 h and 96 h bioassays were not contemporaneous due to feeding of the meals immediately after grinding. Triplicate enclosures were used in an experiment with the AM with 72 h milled SDPB, and five replicate enclosures were used for the AM with 96 h milled SDPB. An unpaired two-tailed *t*-test was used to compare conditions. Error bars on graphed data represent standard error.

### 2.5. Meal Analyses

#### 2.5.1. Engorgement

Masses of individual female mosquitoes were determined in 1 cm diameter expandable feeding capsules to understand how engorgement on the simple AM compared to engorgement on fresh blood and the AM’s components alone. Forty capsules were first weighed empty. Forty mosquitoes were starved of 10% sucrose for 2 h before being knocked down in a freezer for 3 min and added to capsules. Ten capsules each were fed fresh defibrinated bovine blood, the simple AM, 20% SDPB (*w*/*v*) in 1× PBS, or 10% whey (*w*/*v*) in 1× PBS. Capsules with mosquitoes were weighed again before and after the mosquitoes’ 2 h feeding period to obtain the net mass of meal engorged for individual females. Negative calculated masses of meal engorged with an absolute value of less than 10% of the individual mosquito’s measured mass were rounded to zero. One-way ANOVA was used to compare engorgement masses between meal conditions.

#### 2.5.2. Fecundity

To determine whether the inclusion of whey protein in an AM affects fecundity, triplicate negative control enclosures of 20% SDPB (*w*/*v*) in 1× PBS and one positive control replicate (fresh defibrinated bovine blood) were used in an experiment with N = 10 replicates of the simple AM. Feeding was performed for 2 h to maximize engorgement and fecundity. An unpaired two-tailed *t*-test was used to compare the fecundity of the AM to the negative control. Error bars on graphed data represent standard error.

#### 2.5.3. Colony Selection and Egg Viability

Mosquito meals must sustain generational feeding of a colony to be viable for rearing purposes, so an *Ae. aegypti* colony was reared exclusively on the simple AM. Per the procedure described within Section 2.2: General Feeding Bioassay, females 6–15 d old were fed the simple AM for 2 h without starvation in a BugDorm-1© enclosure. This was performed multiple times, if necessary, to gather a quantity of eggs sufficient for hatching. For colonies raised exclusively on the AM, eggs were stored dry after being removed from BugDorm-1© cages for 14 d. Eggs were then hatched to propagate successive generations of an exclusively AM-fed colony. Egg counts per female were measured for each generation and contemporaneously compared to egg counts per female from a similar-age laboratory mosquito colony fed fresh defibrinated bovine blood. Feeding data from F6–F8 generations were not considered due to a time discontinuity between rearing of the F5 and F6 generations that affected the colony’s fecundity.

Larval and pupal hatch rates were evaluated for the F8 generation by aliquoting 400–600 eggs into a deli cup, hatching them in 400 mL of well water (1–1.5 eggs/mL), and then counting the number of larvae and pupae within 20–40 mL aliquots between 0 d and 7 d post-hatch. A minimum of triplicate measurements were taken per condition, and eggs were hatched in two separate enclosures. Error bars on graphed data represent standard error.

#### 2.5.4. Meal Stability

The simple AM and defibrinated bovine blood were fed at 0 d and 14 d post-formulation to determine the effect of aging on meal nutritional value. At 14 d post-formulation, the same aqueous preparation of AM and container of bovine blood were removed from refrigeration and used to feed mosquitoes of approximately the same age as those used in the earlier experiment (±1 day) in the same alternating configuration. Six replicates were used for the initial feeding, and eight replicates were used for the second feeding. An unpaired two-tailed *t*-test was used to compare the two conditions. Error bars on graphed data represent standard error.

To simulate one year of AM aging under ambient conditions, two 10 g preparations of dry simple AM in glass scintillation vials were incubated at 54 ± 2 °C for 14 d, reflecting testing guidelines provided by the U.S. Environmental Protection Agency for pesticide storage stability [33]. Significant Maillard browning of whey protein powder can occur under these conditions and is accelerated by the presence of PBS buffer salts in the simple AM [34]. Elements of the study pertaining to corrosion characterization were not implemented. One 10 g preparation was treated with an N_2_ blanket before incubation, and the other’s headspace was filled with air. Fresh defibrinated bovine blood, nonaged simple AM, and the two aged AM preparations were fed in an alternating configuration in triplicate for each condition. An unpaired two-tailed *t*-test was used to compare the two conditions. Error bars on graphed data represent standard error.

#### 2.5.5. Meal Cost

The simple AM’s component costs was compared to SkitoSnack component costs furnished by Gonzales et al. [12] and market prices set by HemoStat Laboratories [35], an eminent animal blood supplier.

### 2.6. Statistical Analyses

Comparative fecundity trials were used in most feeding experiments and were analyzed with unpaired two-tailed *t*-tests (UTT) with the null hypothesis that fecundity was either not significantly different than a control or not significantly different than zero. One-way ANOVA was performed with engorgement data to test for significance of differences between condition means. The Tukey’s honestly significant difference (HSD) test was applied post hoc for pairwise comparisons. Graphed error on plots represents standard error with standard deviation used only for ATP luminescence and particle diameter measurements.

## 3. Results

### 3.1. Phagostimulant Analyses

#### 3.1.1. SEM/Light Microscopy and Laser Diffraction

**Figure 2:**
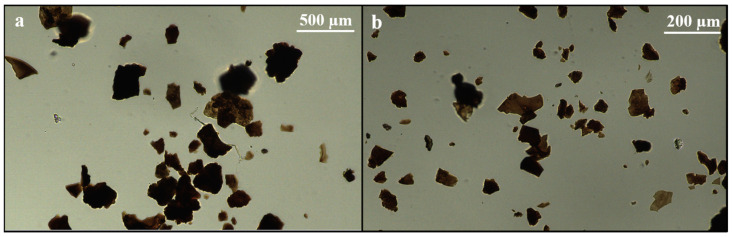
Light microscopy of unground SDPB visualized on Neubauer hemocytometer slides at scale lengths of (**a**) 0.1 mm and (**b**) 0.05 mm.

Understanding of the particle morphology and size distribution of SDPB provides useful information for post-processing methods of the spray-dried product. Visual inspection revealed a broad range of particle types from black semicrystalline masses to fine red–brown dust. Light microscopy of the unground powder revealed amorphous, opaque aggregates of blood components, visible in Figure 2.

**Figure 3:**
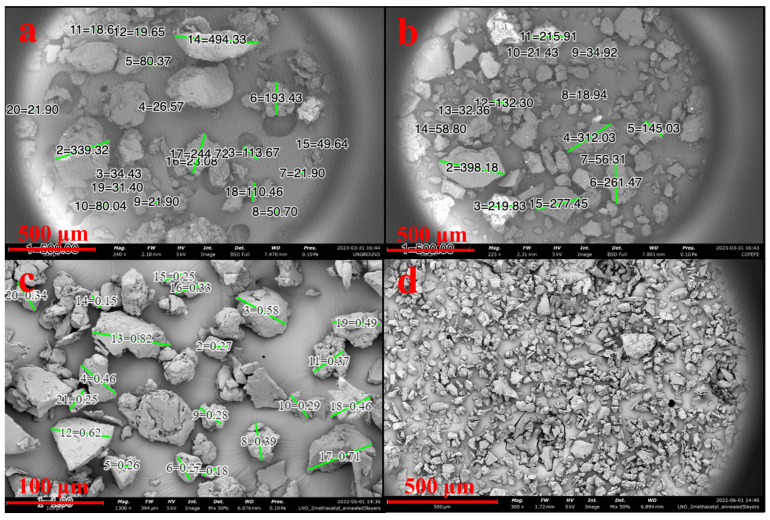
Preparations of SDPB imaged via 5 kV SEM. Subfigures represent SDPB (**a**) unground, (**b**) coffee-ground, (**c**) espresso-ground, and (**d**) espresso-ground and sieved through a 63 µm wire mesh. Green lines in subfigures (**a**–**c**) represent digitally generated particle diameters. Scale lengths of 500 µm or 100 µm are represented by a red bar in each subfigure.

**Figure 4:**
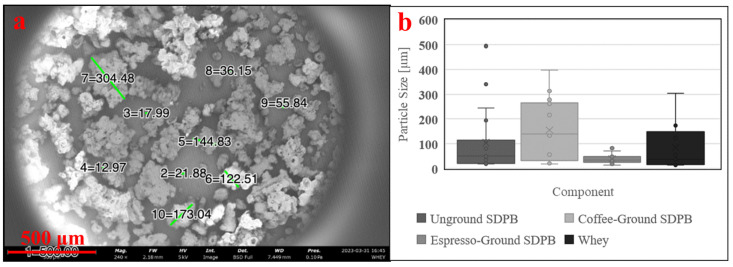
Preparation of whey powder (**a**) imaged via 5 kV SEM and (**b**) box-and-whisker plots of measured particle diameters of SDPB in Figure 3a–c and the whey preparation. Green lines in subfigure (**a**) represent digitally generated particle diameters. A minimum of ten particle diameters were estimated at random for each condition. Error bars represent standard deviation.

SEM showed SDPB particle morphologies with greater contrast in Figure 3. The unground particulate appeared more spherical and oblong, while ground particles showed heavy fragmentation consistent with shear fractures. Measured particle diameters in each SDPB image and whey particulate in Figure 4a were tabulated in Figure 4b with the lowest mean and median diameters attributable to the espresso-grinding technique. Whey was observed to have a relatively large particle diameter distribution with the second-lowest median (36.2 µm) to espresso-ground SDPB (33.5 µm), followed by unground SDPB (49.6 µm) and coffee-ground SDPB (138.7 µm). The range of diameters decreased with the fineness of the grinding technique with unground, coffee-ground, and espresso-ground SDPB spanning 476 µm, 379.2 µm, and 67 µm, respectively, with whey particles spanning 290 µm.

**Figure 5:**
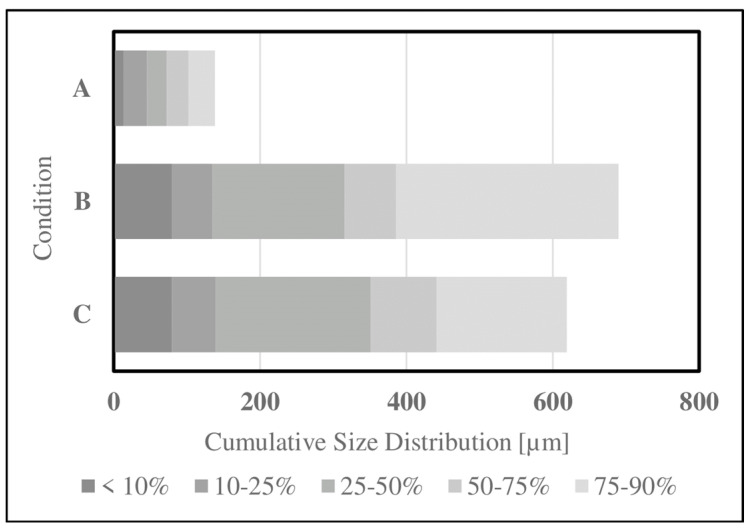
Cumulative particle distribution measurements gathered for (**A**) whey powder, (**B**) coffee-ground SDPB, and (**C**) unground SDPB via laser diffraction.

Particle reduction methods were investigated to improve the accessibility of SDPB in the simple AM. Particle size and distribution data for aqueous SDPB and whey demonstrate a large disparity in the proportion of particles ingestible by a mosquito (20–50 μm) in Figure 5. Unground SDPB demonstrated a mildly right-skewed distribution with a mean diameter of 350.7 ± 339.3 µm. In total, 6.21% of measured particles had diameters below 50 µm. Coffee-ground SDPB demonstrated a smaller mean diameter of 315.7 ± 296.4 µm. Whey measurements produced a mean diameter of 72.2 ± 46.5 µm.

#### 3.1.2. LC–MS/MS Proteomics

**Figure 6:**
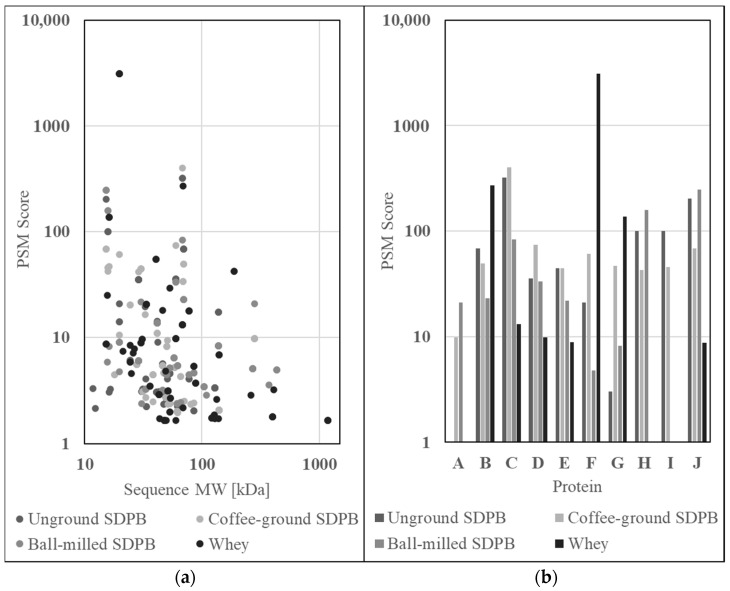
Graphs of LC–MS/MS SDPB and whey protein spectral matches (**a**) by sequence molecular weight and (**b**) for ten proteins of high alignment between all conditions. Proteins included within this subset were (A) filamin A, (B) albumin (bovine or porcine), (C) protease 1, (D) ubiquitin C, (E) apolipoprotein A-1, (F) β-lactoglobulin, (G) α-lactoglobulin, (H) hemoglobin subunit beta, (I) hemoglobin fetal subunit beta, and (J) hemoglobin subunit alpha.

Proteomics data of trypsin-digested SDPB and whey powder were gathered via liquid chromatography–mass spectrometry (LC–MS) to ascertain the nutritional qualities of AM components. Raw abundance scores, provided as protein spectral match scores (PSMs), for the constituents of unground SDPB, coffee-ground SDPB, ball milled SDPB, and whey powder are shown in Figure 6a. Most proteins identified possessed molecular weights of 10–100 kDa with whey powder contributing both the highest-scoring (β-lactoglobulin, 3118.98) and largest (submaxillary apomucin, 1183 kDa) proteins to the assay results. Ball-milled SDPB yielded more 100–1000 kDa protein matches than unground and coffee-ground SDPB with seven, two, and two matches, respectively.

From all protein matches of high alignment, a subset of matches with nutritive properties or large differences in abundance between whey and SDPB were selected and are visible in Figure 6b. Proteins included within the subset are (A) filamin A, (B) albumin (fragment), (C) protease 1, (D) ubiquitin C, (E) apolipoprotein A-1, (F) β-lactoglobulin, (G) α-lactoglobulin, (H) hemoglobin subunit beta, (I) hemoglobin fetal subunit beta, and (J) hemoglobin subunit alpha.

PSMs scoring greater than ten for the three preparations of SDPB and whey powder were ranked from high to low in Table 2. Proteins identified in all four conditions include protease 1 and albumin, while proteins exclusive to the SDPB preparations include hemoglobin (Hb) subunits alpha and beta, apolipoprotein, ubiquitin C, and γ-actin. Both subunits of hemoglobin ranked within the top five most abundant identified proteins in the unground and ball-milled SDPB preparations, while the coffee-ground SDPB preparation featured these proteins within the top ten. Disregarding protease 1 (a likely contaminant), hemoglobin subunit alpha produced scores 100%, 93%, and 100% of the most abundant protein identified.

#### 3.1.3. ATP Assays and Microbial Contamination

**Figure 7:**
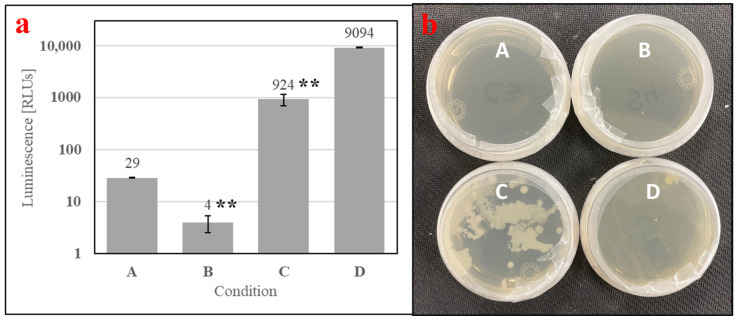
Contamination assay of (A) boiled water and aqueous preparations of (B) autoclaved SDPB, (C) “raw” SDPB, and (D) EC-1118 *S. cerevisiae*, showing (**a**) ATP luminescence measurements and (**b**) agar culture plates. Asterisks indicate significant differences in engorgement between the denoted meals (UTT; ** for *p* < 0.01). Error bars represent standard deviation.

To understand if microbial metabolites were affecting the phagostimulation of SDPB, initial ATP luminescence measurements of aqueous autoclaved and “raw” SDPB (Conditions B and C, respectively), as well as negative (Condition A) and positive (Condition D) contamination controls, were taken preculture and are shown in Figure 7a. Autoclaving of SDPB significantly affected luminescence measurements (UTT t_5_ = 6.55, *p* = 0.0012), reducing RLU intensity to 0.4% of the measurement observed for SDPB not autoclaved.

Exemplary agar replicates post-culture (18 h at 28 °C) demonstrated no visible contamination in the negative control and autoclaved SDPB conditions, while white, splotchy colonies quickly proliferated in Condition C and a film of small *S. cerevisiae* colonies were visible in the positive control in Figure 7b. Consistent results were observed for all replicate plates.

#### 3.1.4. Heat Sterilization

**Figure 8:**
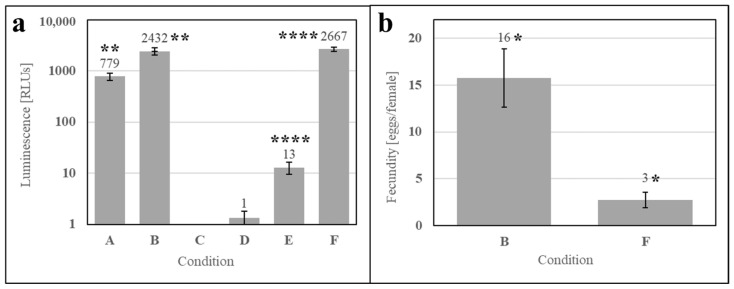
Aqueous culture and feeding of the simple AM after (A) 0 h and (B) 2 h, autoclaved SDPB after (C) 0 h and (D) 2 h, and the simple AM with autoclaved SDPB after (E) 0 h and (F) 2 h. Subfigures show (**a**) ATP luminescence measurements and (**b**) fecundity of the simple AM and the AM containing SDPB autoclaved at 121 °C for 1 h. Asterisks indicate significant differences in engorgement between the denoted meals (UTT; * for *p* < 0.05, ** for *p* < 0.01, **** for *p* < 0.0001). Error bars represent standard deviation in (**a**) and standard error in (**b**).

To evaluate whether the phagostimulant was a metabolite of contaminants in SDPB, ATP luminescence measurements were performed pre- and post-culture (2 h) for the simple AM (Conditions A and B, respectively), an aqueous solution of autoclaved SDPB (Conditions C and D), and the simple AM with autoclaved SDPB (Conditions E and F). Figure 8a shows significant increases between ATP measurements at 0 and 2 h for the simple AM (UTT t_5_ = 6.69, *p* = 0.0011) and the simple AM with autoclaved ATP (UTT t_5_ = 16.83, *p* < 0.0001). A significant difference in fecundity was observed between these two conditions UTT t_5_ = 2.40, *p* < 0.013) with the AM containing autoclaved SDPB yielding a fivefold reduction in fecundity when compared to the simple AM in Figure 8b.

#### 3.1.5. Ball Milling

**Figure 9:**
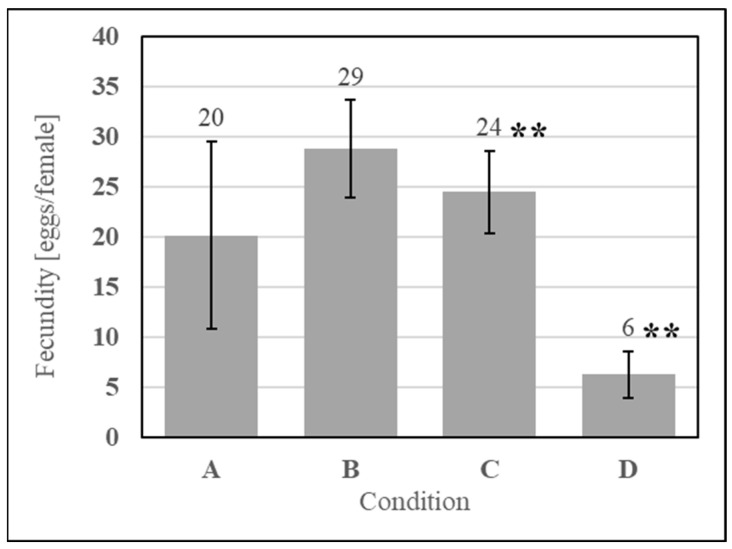
Feeding of AMs containing ball-milled SDPB with fecundity of the simple AM (A/C) and AMs containing SDPB ball-milled for 72 h (B) and 96 h (D). Asterisks indicate significant differences in engorgement between the denoted meals (UTT; ** for *p* < 0.01). Error bars represent standard error.

Feeding of SDPB ball-milled for 72 h and 96 h (in the simple AM) was performed to observe the effect of particle reduction on fecundity. Milling SDPB for 72 h increased fecundity by 45% relative to coffee-ground SDPB, while milling for 96 h significantly reduced fecundity by 75% (UTT t_8_ = 3.90, *p* = 0.0045), as shown in Figure 9.

### 3.2. Meal Analyses

#### 3.2.1. Engorgement

**Figure 10:**
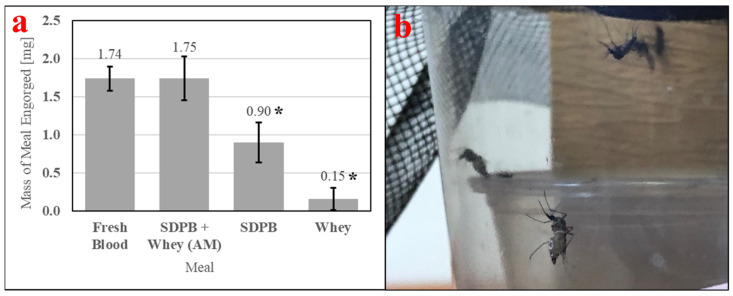
Engorgement of individual mosquitos with (**a**) mass measurements of engorgement on fresh bovine blood, the simple AM, 20% SDPB (*w*/*v*) in PBS, and 10% whey (*w*/*v*) in PBS and (**b**) female *Ae. aegypti* fully engorged on SDPB. Asterisks indicate significant differences in engorgement between the denoted meals (ANOVA; * for *p* < 0.05). Error bars represent standard error.

Feeding of *Ae. aegypti* is enabled by consistently high engorgement of a meal by individual mosquitoes. Therefore, it is important to measure in feeding bioassays. Table 3 contains ANOVA conducted with engorgement mass data and Tukey’s HSD test applied post-hoc. Figure 10a quantitatively demonstrates ingestion of the simple AM, which is visible in Figure 10b. A significant difference in engorgement mass was observed between meals (ANOVA F_3,36_ = 10.58, *p* < 0.0001). Engorgement on the simple AM was not significantly different compared with engorgement on fresh bovine blood (UTT t_18_ = 0.0003, *p* = 0.99), while engorgement on SDPB alone was significantly different compared with engorgement on whey alone when analyzed with UTT (t_18_ = 5.46, *p* = 0.031) but not with Tukey’s HSD test (*q* = 3.17, *p* = 0.13). The mean mass of meal imbibed upon was approximately twice as large for fresh blood and the simple AM relative to SDPB, while the mean mass of SDPB ingested was six times larger than the mean mass of whey ingested.

#### 3.2.2. Fecundity

**Figure 11:**
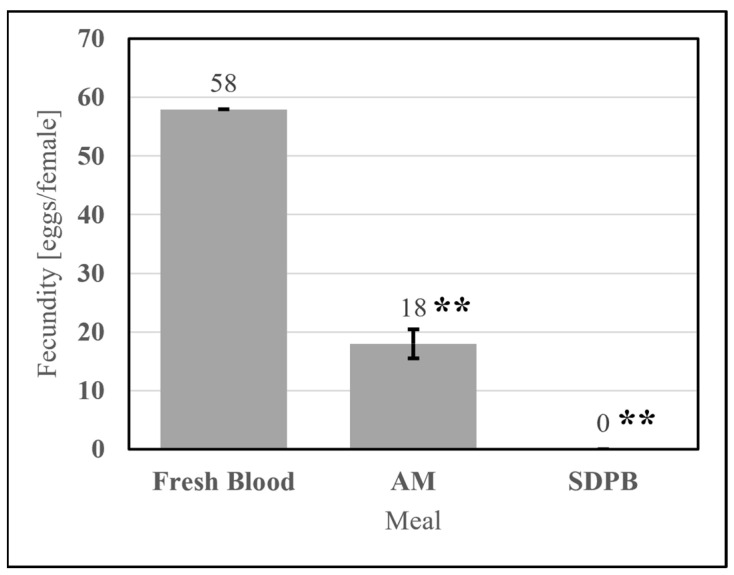
Fecundity of fresh bovine blood, the simple AM, and 10% whey in PBS meals. Asterisks indicate significant differences in engorgement between the denoted meals (UTT; ** for *p* < 0.01). Error bars represent standard error.

Meals useful for mosquito rearing must stimulate consistently nonzero egg production. The simple AM stimulated egg production significantly greater than that of 20% SDPB (*w*/*v*) in PBS (UTT t_11_ = 3.88, *p* = 0.0026). SPDB alone failed to stimulate oogenesis in all replicates tested, as shown in Figure 11, indicating that the addition of whey powder to the spray-dried blood powder enabled oogenesis. On average, *Ae. aegypti* imbibing upon the simple AM produced 31% of the egg count of *Ae. aegypti* imbibing upon fresh defibrinated bovine blood.

#### 3.2.3. Colony Selection and Egg Viability

**Figure 12:**
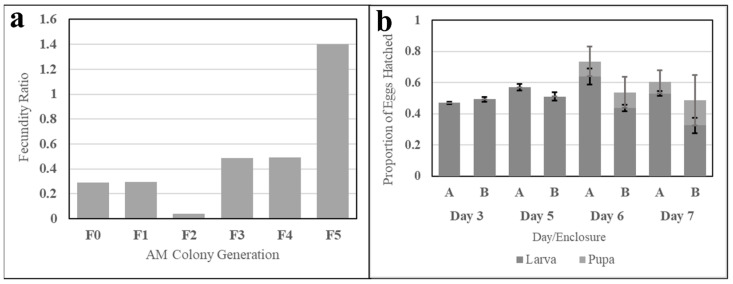
Fecundity and egg viability evaluations of *Ae. aegypti* reared exclusively on the AM. Subfigure (**a**) shows ratios of six generations of an *Ae. aegypti* colony exclusively fed the simple AM. The ratio represents AM-colony egg fecundity relative to the fecundity of a fresh-blood-fed sister colony. Subfigure (**b**) shows larval and pupal hatch ratios relative to the number of eggs aliquoted for hatching. Data from clutches (A) and (B) are grouped by day post-hatch. Error bars represent standard error.

The fecundity of six successive generations of simple AM-fed *Ae. aegypti* was evaluated to determine the capacity of the meal to sustain a colony (necessary in mass rearing) and demonstrates a positive upward trend. The first AM-fed, or F0, generation produced 28% of the egg productivity of a sibling colony fed fresh bovine blood, and the fecundity ratio of successive generations increased (discounting the F3 generation), as shown in Figure 12a.

Larval and pupal hatch rates were evaluated for two clutches of the eighth-generation AM-fed colony. The proportions of larvae and pupae to the initial egg count from three to seven days post-hatch are shown in Figure 12b. Larval count increased until Day 6 for Enclosure A and Day 5 for Enclosure B, while pupal emergence created a significant drop-off in larval counts during Days 6 and 7. Larval hatch for each enclosure was observed to exceed 50%.

#### 3.2.4. Meal Stability

**Figure 13:**
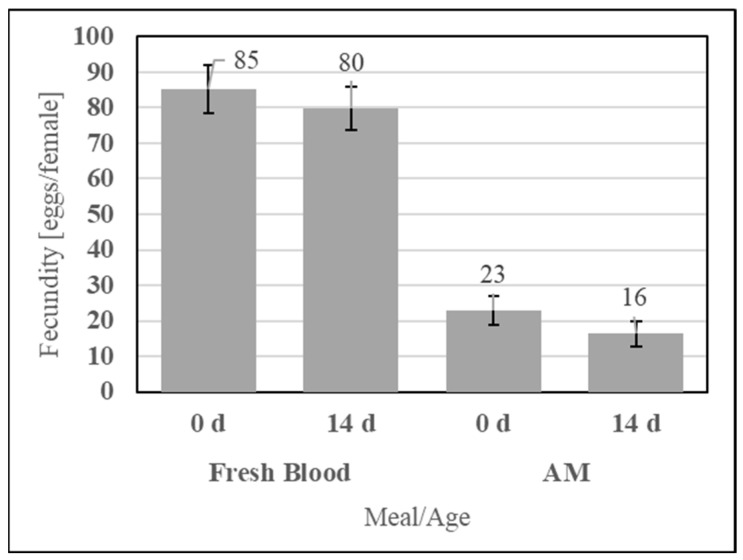
Fecundity of fresh blood and simple AM meals when fed immediately (0 d) and 14 d post-collection for fresh blood or post-preparation for the AM. Error bars represent standard error.

To evaluate the feeding efficacy of the aqueous simple AM compared to the storage life of fresh defibrinated bovine blood, the two meals were fed at 0 d and 14 d of post-preparation age, as shown in Figure 13. No significant differences in fecundity were observed for fresh blood (UTT t_12_ = 0.59, *p* = 0.58) or the AM (UTT t_12_ = 1.19, *p* = 0.26) between feeding at 0 d and 14 d.

**Figure 14:**
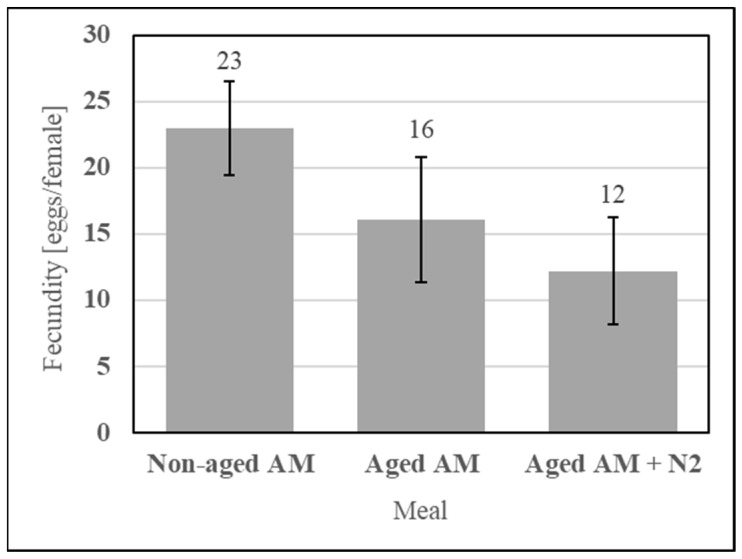
Fecundity data obtained from feeding the simple AM with the dry and sealed formulation not aged, aged, or aged with an N_2_ blanket. Error bars represent standard error.

Dry stability of the simple AM enables a lot of meal to be fed many times, which positively affects repeatability. It was evaluated using an accelerated aging protocol simulating one year of ambient storage consisting of incubating the sealed formulation at 54 °C for 14 d. The simple AM components (SDPB, whey powder, and PBS salts) were incubated with or without an N_2_ blanket and fed alongside a nonaged AM control, as shown in Figure 14. Neither aging under a normal atmosphere (UTT t_4_ = 0.95, *p* = 0.40) nor aging under an N_2_ blanket (UTT t_4_ = 0.51, *p* = 0.64) significantly affected fecundity relative to the nonaged AM.

#### 3.2.5. Cost Evaluation

A table of costs per liter by component of the simple AM, SkitoSnack, and fresh bovine blood is described in Table 4. Component costs for SkitoSnack and defibrinated bovine blood reflect the values reported by Gonzales et al. [12] and HemoStat Laboratories [35].

The material cost of the simple AM was reduced by 50% by including the ionic constituents of PBS as salts in a dry mixture, which allowed for simplified packaging of the meal as a dry powder and reconstitution with deionized water.

## 4. Discussion

### 4.1. Phagostimulant Function and Nutrition

As a spray-dried powder comprising desiccated erythrocytes, proteinaceous macromolecules, and various plasma components, the biochemical functions of SDPB in mosquito feeding are complex. Particle distribution measurements of unground SDPB in Figure 5 indicate that the standard deviation of particle diameters measured (339.3 µm) nearly eclipsed the mean diameter (350.7 µm), underscoring the large variance in diameters observed in the raw material. SEM (Figure 3) and light microscopy (Figure 2) showed that manual grinding methods and sieving can select for particles ingestible through the mosquito proboscis, or roughly 20–50 µm [36]. Yet, despite some accessibility of small SPDB particles, the imbibed meal appeared white in the mosquito midgut, indicating that, primarily, whey was ingested by *Ae. aegypti* as albumin is approximately 7.5 × 6.5 × 4 nm in size [37]. Little visible ingestion of SDPB implies that that many proteins in fresh blood denature and aggregate when spray-dried, becoming too large to ingest; however, if a protein, the phagostimulant must be ingestible when solubilized.

Ten relatively abundant proteins identified via LC–MS/MS proteomics in SDPB are shown in Figure 6b. From this list, only hemoglobin (Hb) subunits α and β were far more abundant in SDPB than in whey and are also known to positively affect mosquito feeding and nutrition [8,9,12]. Characterization of the mechanisms of whole blood droplet drying indicate that some Hb function may be preserved through the spray drying process. As water evaporates from droplets, the high degree of advective heating applied to its surface coupled with low initial mass diffusion produces a Marangoni effect of solute migration via surface tension [16], which is characteristic of a high Peclet number and diffusion hindering the movement of protein molecules towards the droplet interior as the evaporating surface recedes [16,19,38]. This overrepresentation of protein at the droplet surface has also been characterized with the spray drying of trypsin and bovine serum albumin [26,27]. The dried particle aggregate is a hollow, porous shell of monomers held together by hydrophobic interactions [21], which SDPB spherical structures observed in Figure 3 could plausibly represent due to their size (<50 µm) and hollow/dimpled geometries.

Spray drying is not an inherently sterilizing process due to the transient and relatively low wet-bulb temperatures predictable using Equation (1), precluding hemoglobin from being the only possible phagostimulant in the AM. While achieving significant reductions in concentrations of key food contaminants including *Salmonella* and *E. faecium* (up to log 4.15 and 2.83 reductions, respectively), Steinbrunner et al. found microbes in all places sampled within a pilot-scale spray-drier used to desiccate soy protein isolate and determined that modifying inlet gas temperatures had no significant effect on microbial inactivation [39]. Accordingly, significant contamination of SDPB used in the present experimentation is evident in Figure 7 with rapid and visible growth observed from solubilized “raw” SDPB. Microbes in whey contributed to significant growth within the prepared AM with autoclaved SDPB over the course of a feeding period (2 h) when compared to aqueous autoclaved SDPB alone (Figure 8).

There is some evidence that yeast products in insect meals, particularly fruit flies, supplement nutrition by providing additional protein, nutrients, and/or phagostimulants [40,41,42]. However, it is unlikely that contaminating yeast is affecting feeding on the AM given that ATP luminescence measurements of the simple AM and AM with autoclaved SDPB were not significantly different after 2 h in solution and fecundity was significantly lower in the latter meal (Figure 8). Autoclaving SDPB at 121 °C for 1 h significantly impacted both ATP measurements and fecundity, indicating that the denaturation of accessible hemoglobin may decrease feeding. To this end, it is unlikely that any microbes in SDPB produce a phagostimulant with a greater nutritional effect than that of hemoglobin.

### 4.2. Improving SDPB Attributes

The large numbers of replications performed within each feeding experiment were a necessary consequence of the feeding behavior of *Ae. aegypti* on the AM. Among mosquitoes in individual enclosures, engorgement on all AMs was highly variable, requiring the use of N = 50–100+ females across multiple cages to manage errors introduced by inconsistent feeding, slight temperature decreases across the feeding chain, and environmental variations like humidity and light exposure. Other techniques included standardizing Parafilm^®^ stretching, thicknesses, and application of hand oils (which act as phagostimulants), as well as feeder pressures on enclosures and the volume of AM within feeders. The repeatability of feeding is an important characteristic of insect meals, so many efforts were made to increase the accessibility of SDPB by particle size reduction.

Mechanical grinding was identified as the simplest and most accessible means of processing hemoglobin in SDPB to improve egg fecundity. Automated milling of the powder for 72 h and 96 h yielded significantly better and poorer fecundity than simple AM preparations (Figure 9), respectively, indicating that there may be a “sweet spot” for particle size that improves Hb accessibility without (1) reducing a significant portion of non-nutritive particles to 10–50 µm, thereby impeding feeding; (2) creating excess heat via friction that denatures Hb; or (3) increasing the amount of accessible Hb enough to suppress the feeding response. Milling techniques are typically used for dry powders that do not have the tendency to agglomerate in solution [24], so it is possible that increased aqueous concentrations of small particulate increased aggregation upon solubilization. Future studies on the ball milling of SDPB should characterize precise particle size distributions to understand whether proboscis-clogging particulate, denaturation, or an overabundance of Hb may be affecting feeding for longer SDPB milling times.

### 4.3. AM Performance

Critical attributes of useful alternative meals for *Ae. aegypti* are visible, consistent engorgement, good egg productivity, and nutrition sufficient for an alternative meal-fed colony to adapt to the meal over multiple generations. A simple AM formulation with spray-dried porcine blood and whey protein powder in PBS achieved 31% of the fecundity of fresh blood, or 18 eggs/female, with *Ae. aegypti* engorging upon both the fresh and alternative meals similarly (Figure 10a). Most feeding assays evaluating the simple AM yielded fecundity ratios of 25–35%. Assuming an average of 3500 females per cage (1:1 sex ratio), Tyler-Julian et al. observed a mean egg productivity of 40 ± 4 eggs/female/month for the same strain of *Ae. aegypti* when feeding frozen, citrated bovine blood weekly [11], or approximately 10 ± 1 eggs/female/feeding if four feedings are performed per month. The egg productivity of Orlando-strain *Ae. aegypti* was nearly twice as great when feeding the AM versus frozen blood by this analysis. Tyler-Julian et al. note that their feeding methods successfully sustained colonies of *Ae. aegypti* for 2 y. At such levels, both mass- and laboratory-scale rearing are feasible but offer room for improvement relative to fresh blood and other AMs.

Comparative feeding of six generations raised on this AM demonstrated increasing fecundity relative to fresh blood generation-over-generation, nearing 50% (Figure 12a). Adaptation of the AM-fed colony to the simple meal yielded increasing egg productivity ratios around 48% for the F3 and F4 generations after a dip to 4% for the F2 generation, which may be due to a selection bottleneck caused by AM nutrition or feeding methods as compared to live host feeding; Ross et al. observed inbreeding depression with artificial membrane-selected *Ae. aegypti* populations [43]. As a general trend, increasing egg productivity ratios across colony generations may positively be attributed to adaptation, cited by Kandel et al., who observed that a Ugal-strain *Ae. aegypti* colony exclusively fed SkitoSnack demonstrated an egg production ratio increase from 84% for the F0 generation to 100% for the F30 generation [9]. Tyler-Julian et al. did not compare the performance of frozen blood to fresh blood, nor did they rear *Ae. aegypti* used in direct experimentation past the F0–F4 generations, rendering adaptation comparisons between frozen blood and the AM difficult to construct. For the AM-fed colony, data from the F5 generation appear to be outlying and could be due to issues with the quality of fresh blood fed. A change in the supplier of SDPB after the F5 generation coupled with incubator faults experienced during rearing of the F6 and F7 generations disqualify their data from direct comparison with previous generations.

Larval hatch and pupation for the F8 generation were lower than that observed for blood-feeding *Ae. aegypti* with a maximum larval/pupal combined ratio of 74% observed at 6 d post-hatch for the first enclosure measured (Figure 12b). Hatch rates ≥ 90% can be expected from both fresh and frozen blood feedings of the wild-type with pupation rates around 90% [9,11,44,45], which differs noticeably from rates observed for the F8 generation. The observed time to eclosion of <3 d (emergence of larva from eggs), however, was similar to established emergence times of ~2 d [11,44,45]. Disparities between the hatch-to-adult time (7+ d for the AM colony), which is typically 5–7 days for a blood-fed line [44], could be indicative of inadequacies in the nutritional profile of the simple AM formulation as it has not been validated as nutritionally complete.

Several limitations constrain the interpretation of feeding results obtained in the present study. First, the generated data can only predict feeding performance when starvation, feeding, and egg oviposition-to-hatch times are followed as prescribed for 10–20 female *Ae. aegypti* mosquitoes. Larger or smaller quantities of *Ae. aegypti* fed, starved, and hatched under differing periods may feed and oviposit differently. The reproducibility of fecundity data increases drastically with increasing mosquito sample sizes as a fraction of female *Ae. aegypti* fully engorged on the simple AM. Ambient temperature and relative humidity can also drastically impact mosquito engorgement and oogenesis [46], which were controlled for experimental reproducibility.

### 4.4. AM Cost and Stability

At a material expense of under USD 10 per liter of aqueous meal, the simple AM formulation costs less than 1/8th of fresh defibrinated bovine blood and 1/20th of SkitoSnack on a volumetric basis (Table 4) while providing 25–35% of the egg productivity of fresh bovine blood. Replacing aqueous PBS with its constituent phosphate and sodium salts in dry form reduces the material cost of the simple AM to less than USD 5 per liter. Existing AMs and fresh bovine blood require refrigeration, adding to their costs of use, and fresh bovine blood is handled, stored, and disposed of as a biohazard, increasing labor and material expenses. SDPB AMs can be stored dry at room temperature and may be prepared with basic laboratory equipment (a graduated cylinder, pipette, magnetic stirrer, beaker, and a balance) in less than 10 min.

An important attribute of an alternative meal is its shelf life. Long shelf lives enable the bulk formulation of a single lot of an AM, reducing transportation and storage costs while homogenizing the mosquito feeding response for precise experimentation. Dry, refrigerated SkitoSnack is reported to retain feeding efficacy within 50 ± 5 eggs/female for 84 d [12] as it contains cold-stored ingredients with short expiration times (i.e., bovine serum albumin). The usable storage life of SDPB is uncharacterized due to its lack of applications outside of animal feedstocks but has been observed to be at least eight months once packaging is breached. The fecundity of feeding the simple AM at 14 d post-preparation is not significantly poorer relative to immediate feeding (Figure 13), while mosquito engorgement on a hydrated preparation of SkitoSnack significantly decreases after 4–5 h [12]. Regarding the other simple AM components, processed whey powders and PBS formulations are often shelf-stable for multiple years. The present results imply that spray-dried Hb is relatively stable in the aqueous AM and can be stored dry for the duration of many feeding experiments (1+ y).

These cost and stability attributes of the AM provide multiple benefits to mass rearing strategies. The increasing egg productivity ratios observed with successive AM-fed colony generations reduces the amount of meal required for feeding and improves egg yield, providing a cost benefit valuable to vector control wherein millions of irradiated males are needed on a monthly basis [47,48], for example. The consistency of rearing practices and materials is paramount to obtaining quality males [47] and may be aided by the ambient stability of the AM as a single lot can be used to feed a colony for a long period of time. In general, the formulation’s lack of biohazardous components may improve the efficiency of mass rearing by obviating safety and administrative controls surrounding the handling of fresh blood in large volumes.

## 5. Conclusions

Challenges arising from the use of fresh animal blood for rearing mosquitoes stymies progress towards the eradication of vector-borne diseases. Spray-dried porcine blood (SDPB) alternative meals (AMs) present a promising feeding platform compared to fresh bovine blood in the rearing of *Aedes aegypti* mosquitoes. Biochemical and material analyses of SDPB support the hypothesis that the shelf-stable phagostimulant is most likely hemoglobin aggregates preserved through spray drying and not a microbial metabolite. However, SDPB stimulated oogenesis in *Ae. aegypti* only when coupled with a source of accessible albumin, and the nutritive capacity of spray-dried hemoglobin is not fully understood. Automated mechanical grinding is a potential post-processing route for improving the accessibility of solubilized hemoglobin by affecting AM fecundity. Future work may also examine reassembly mechanisms of hemoglobin in spray-dried whole blood and subsequent insect nutritional bioassays.

SDPB within a simple meal formulation demonstrated use by stimulating high levels of engorgement and sustaining nine generations of a colony while providing cost and stability advantages over fresh blood. When coupled with a source of ingestible albumin, AMs containing spray-dried blood were fecund at 25–35% of the egg productivity of fresh bovine blood, the expensive and refrigerated meal standard. Multiple generations of *Ae. aegypti* reared exclusively on the AM demonstrated increased egg production and a level of nutrition sufficient to achieve good egg hatch rates. The meal required a feeding time of 2 h while fresh blood typically required 15 min; yet, the meal is not biohazardous, is stable wet and dry, can be prepared quickly and without caution, and costs less than 1/8th that of fresh defibrinated bovine blood (at USD 82/L) and an even smaller fraction of existing AMs.

An alternative meal formulation of spray-dried porcine blood and whey powder in phosphate buffer solution served as an inexpensive, stable, and potentially useful tool for research purposes and mass rearing. The eventual removal of biological and cost-related barriers to the feeding of hematophagous insects on fresh blood will contribute positively to the eradication of vector-based diseases worldwide.

## Figures and Tables

**Table 1:** Description of simple AM preparation and components.

Component	Manufacturer	Concentration	Preparation
10× phosphate-buffered saline	Thermo Scientific Inc. (Waltham, MA, USA)	Diluted in 9 parts DI water, 1 part 10× PBS	Prepared first.
Spray-dried porcine blood (SDPB)	Earthworks Health LLC (Norfolk, NE, USA); Tyson Ingredient Solutions (Tyson Foods, Inc.; Springdale, AR, USA)	20% (*w*/*v*) in 1× PBS	Coffee-ground. Added after PBS is diluted.
Whey protein powder, unflavored	Jarrow Formulas (Sherman Oaks, CA, USA)	10% (*w*/*v*) in 1× PBS	Added after SDPB.

**Table 2:** Protein spectral matches (PSMs) with scores above 10 for SDPB preparations and whey via LC–MS/MS proteomics. The originating species (OS) of each PSM is provided with the protein of interest.

	Unground SDPB		Coffee-Ground SDPB		Ball-Milled SDPB		Whey	
Rank	Protein	Score	Protein	Score	Protein	Score	Protein	Score
1	Protease 1 OS = *A. lyticus*	321.35	Protease 1 OS = *A. lyticus*	401.92	Hemoglobin subunit alpha OS = *B. taurus*	246.04	Beta-lactoglobulin OS = *B. taurus*	3118.98
2	Hemoglobin subunit alpha OS = *B. taurus*	203.16	Ubiquitin C OS = *S. scrofa*	73.73	Hemoglobin subunit beta OS = *B. taurus*	158.81	Albumin OS = *B. taurus*	271.43
3	Hemoglobin fetal subunit beta OS = *B. taurus*	99.95	Hemoglobin subunit alpha OS = *B. taurus*	68.57	Protease 1 OS = *A. lyticus*	82.95	Alpha-lactalbumin OS = *B. taurus*	136.63
4	Hemoglobin subunit beta OS = *B. taurus*	99.7	Beta-lactoglobulin OS = *B. taurus*	61	Ubiquitin C OS = *S. scrofa*	33.37	Uncharacterized protein OS = *B. taurus*	55.11
5	Albumin (Fragment) OS = *S. scrofa*	68.44	Albumin (Fragment) OS = *S. scrofa*	49.29	Albumin (Fragment) OS = *S. scrofa*	22.92	Complement C3 OS = *B. taurus*	42.54
6	Apolipoprotein A-I OS = *B. taurus*	44.51	Alpha-lactalbumin OS = *B. taurus*	46.81	Apolipoprotein A-I OS = *B. taurus*	21.8	Vitamin D-binding protein OS = *B. taurus*	29.39
7	Ubiquitin C OS = *S. scrofa*	35.63	Hemoglobin fetal subunit beta OS = *B. taurus*	45.72	Filamin A, alpha OS = *S. scrofa*	20.91	Transthyretin OS = *B. taurus*	24.91
8	Tropomyosin 3 OS = *S. scrofa*	35.2	Apolipoprotein A-I OS = *B. taurus*	44.3	Actin, gamma 1 OS = *S. scrofa*	13.66	Zinc-alpha-2-glycoprotein OS = *B. taurus*	20.59
9	Beta-lactoglobulin OS = *B. taurus*	20.97	Hemoglobin subunit beta OS = *B. taurus*	42.45			Alpha-1-antiproteinase OS = *B. taurus*	18.05
10	Tropomyosin beta chain OS = *B. taurus*	19.63	Tropomyosin 3 OS = *S. scrofa*	41.71			Serotransferrin OS = *B. taurus*	17.77
11	Alpha1 chain of type I collagen OS = *S. scrofa*	17.3	Albumin OS = *B. taurus*	34.02			Protease 1 OS = *A. lyticus*	13.19
12	Actin, gamma 1 OS = *S. scrofa*	14.36	Trypsin OS = *S. scrofa*	20.4				
13	Carbamoyl-phosphate synthetase 1 (Fragment) OS = *S. scrofa*	14.11	Tropomyosin beta chain OS = *B. taurus*	16.57				
14			Actin, gamma 1 OS = *S. scrofa*	10.99				
15			Carbamoyl-phosphate synthetase 1 (Fragment) OS = *S. scrofa*	10.64				

**Table 3:** One-way ANOVA comparing differences in mass of meal engorged (mg) between fresh blood, SDPB + whey (AM), SDPB, and whey (*** for *p* < 0.01, **** for *p* < 0.0001) with Tukey’s HSD test used for pairwise comparisons.

Condition	Meal Mass (Mean ± SE)	ANOVA	Tukey HSD *p*			
		df, *F*, *p*	Fresh Blood	SDPB + Whey (AM)	SDPB	Whey
Fresh Blood	1.74 ± 0.16	3 10.58 ****	0	1	0.073	***
SDPB + Whey (AM)	1.75 ± 0.29			0	0.071	***
SDPB	0.90 ± 0.27				0	0.13
Whey	0.15 ± 0.14					0

**Table 4:** Meal costs per liter of the simple AM, SkitoSnack [12], and fresh defibrinated bovine blood [35] with component costs.

Diet	Component	Price/Unit	Cost
Simple AM	Spray-dried porcine blood	USD 2.64/kg	USD 0.53
	Whey protein powder	USD 39.60/kg	USD 3.60
	Thermo-Fischer 10X PBS	USD 53.00/L	USD 5.30
	Total per liter		USD 9.79
SkitoSnack	Bovine serum albumin	USD 0.89/g	USD 178.40
	Bovine hemoglobin	USD 0.53/g	USD 2.63
	Chicken yolk	USD 0.48/g	USD 2.39
	Adenosine triphosphate	USD 19.04/g	USD 31.48
	Sodium chloride	USD 0.04/g	USD 0.36
	Sodium bicarbonate	USD 0.11/g	USD 0.21
	Potassium chloride	USD 0.162/g	USD 0.05
	Calcium chloride	USD 0.104/g	USD 0.03
	Magnesium chloride	USD 0.053/g	USD 0.01
	Total per liter		USD 215.54
Fresh Bovine Blood	Total per liter		USD ~82

## Data Availability

All data presented in this study are provided within the article and included data sheets. Inquiries may be directed to the corresponding author.

## Supplementary Materials

The following supporting information can be downloaded at: https://www.mdpi.com/article/10.3390/insects15090716/s1, Table S1: LC elution media concentrations of B (%) and flow rates with respect to time.
