# Supplementary material for: Characteristics of a Spray-Dried Porcine Blood Meal for Aedes aegypti Mosquitoes
Source: Insects. 2024 Sep 19;15(9):716. doi: 10.3390/insects15090716 (PMC11432713; doi:10.3390/insects15090716)
Supplement: Supplementary file 1 [file insects-15-00716-s001.zip › insects-3183969-supplementary.pdf]

Table *S1*. LC elution media concentrations of B (%) and flow rates with respect to time.

Time (min)	% B	Flow Rate (nL/min)
0	1	750
3	1	750
15	5	750
15.1	5	300
100	20	300
123	45	300
130	95	300
135	95	300
135.1	1	300
150	1	300
